# In This Issue: Volume 117, Issue 1, January 2026

**DOI:** 10.1111/cas.70303

**Published:** 2026-01-06

## A Combinatorial Effect of Immune Checkpoint Inhibitors and CD40 Agonistic Antibody in Murine Pancreatic Cancer Model

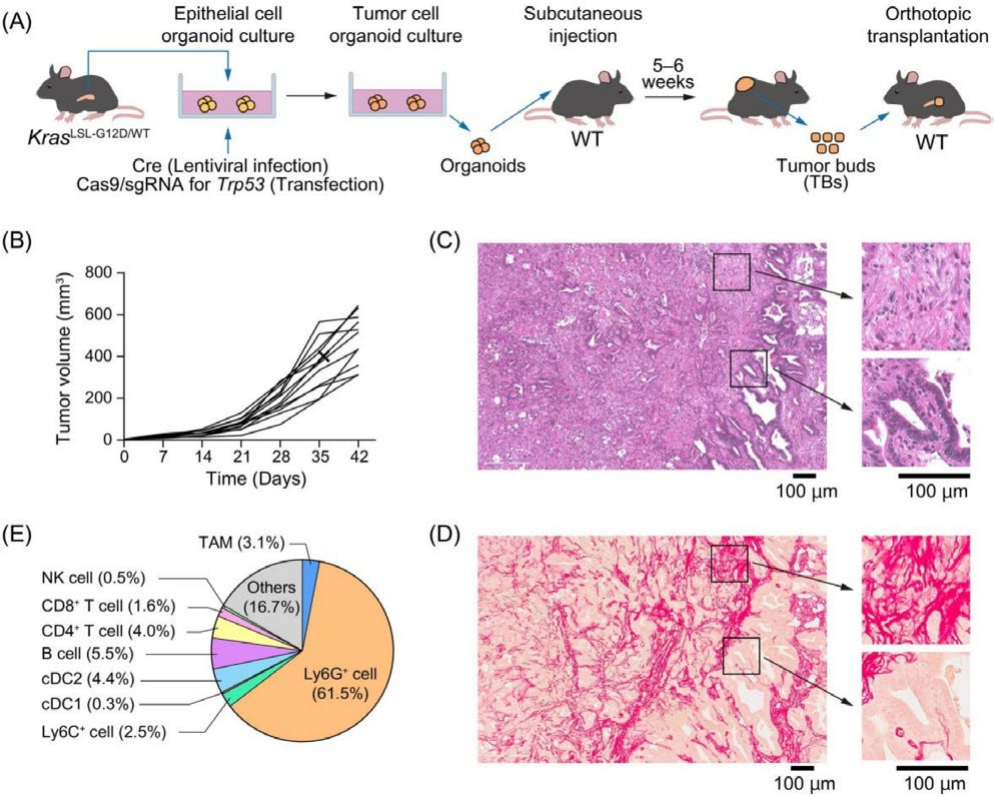

Pancreatic ductal adenocarcinoma (PDAC) remains one of the most lethal forms of cancer, with a five‐year survival rate of only 13%. A major hurdle in treating PDAC is that the tumors are immunologically “cold.” They build a fortress of dense scar tissue (fibrosis) that blocks the immune system from entering, rendering modern breakthrough drugs like immune checkpoint inhibitors (ICIs), which work wonders in cancers like melanoma, largely ineffective for the patients. ICIs treat cancers by blocking certain proteins that function like “brakes” on the immune cells.

To overcome this, Ichikawa et al. engineered a sophisticated new mouse model using genetic engineering approaches. Two genes commonly associated with PDAC, *Kras* and *Trp53*, were targeted. The *Kras* gene was mutated, and the *Trp53* gene was also mutated in the mouse model. Unlike previous models, the new version accurately recreates the disease characteristics and the strong resistance to immune checkpoint blockade seen in patients, providing a realistic platform to test new therapeutic drugs and study the underlying mechanisms involved in the disease progression.

The study combined standard ICIs (which remove the “brakes” on T cells) with a CD40 agonist antibody. This antibody activates antigen‐presenting cells by stimulating the CD40 pathway. The CD40 agonist antibody had only modest effects when used by itself. The combination was more powerful in treating PDAC in the newly developed mouse model. The tumors shrank significantly, mice lived longer, and the immune environment within the tumors shifted from “cold” and suppressive to “hot” and active. The therapy improved the migration of the immune system cells called the dendritic cells to the lymph nodes, enhanced T cell activation, reduced immunosuppressive signals, and even decreased markers associated with tumor‐promoting macrophages.

Overall, their study suggests that combining CD40 activation with checkpoint blockade could be a promising new strategy for patients who currently have few therapeutic options.

https://onlinelibrary.wiley.com/doi/full/10.1111/cas.70246

## A Robust Immunohistochemistry‐Based Classification for BRAF V600E‐Mutant Colorectal Cancer With Clinical Implications

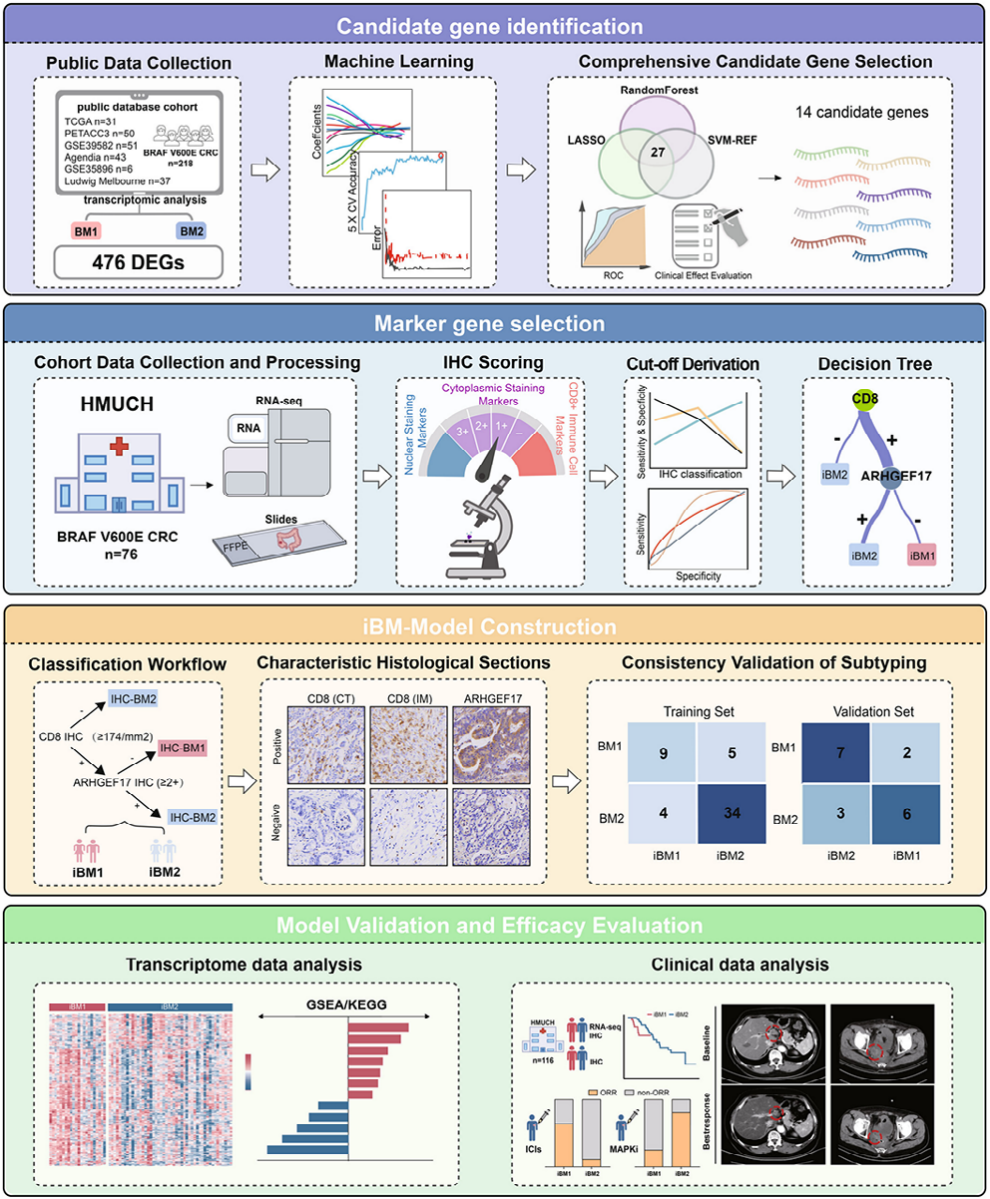

Precision medicine is rapidly replacing the traditional “one size fits all” approach in cancer care. Specific changes in genes—called mutations—can help predict how a cancer behaves and which treatments are likely to work. However, the genome‐sequencing tests needed to identify these differences are costly, time‐consuming, and not routinely available in most hospitals.

Colorectal cancer (CRC) is a major cancer of the large intestine, and about 5%–10% of cases carry a mutation in the *BRAF* gene, most commonly *BRAF* V600E. Even among tumors with this same mutation, behavior can differ depending on which genes are active. Earlier research classified these tumors into two groups: BM1, marked by a process called epithelial–mesenchymal transition (EMT) that helps cancer cells spread and by strong immune activation; and BM2, characterized by increased cell‐cycle activity and altered metabolism. These groups respond differently to treatment, with BM1 generally having poorer outcomes.

Researchers at Harbin Medical University in China aimed to reproduce this useful classification in a simpler way using immunohistochemistry (IHC)—a standard, widely available laboratory technique. From an initial list of 476 genes known to differ between BM1 and BM2, the team used machine‐learning and statistical approaches to narrow the list to two key proteins: CD8, an immune marker, and ARHGEF17, a protein involved in cell‐cycle regulation. Based on the patterns of these two markers, tumors were grouped into iBM1 and iBM2.

The new IHC‐based system showed over 70% agreement with the original gene‐expression classification across multiple patient groups. Tumors classified as iBM1 displayed EMT‐related and immune‐active features, had poorer survival, and responded better to immune checkpoint inhibitors (ICIs). In contrast, iBM2 showed features of rapid cell division and appeared to benefit more from treatments targeting the BRAF pathway, such as MAPK and EGFR inhibitors.

Overall, this new IHC‐based method provides a practical, accessible way to classify *BRAF* V600E‐mutant colorectal cancers. Because it uses routine pathology techniques, it could be widely adopted to help guide treatment decisions and improve personalized cancer care.

https://onlinelibrary.wiley.com/doi/full/10.1111/cas.70231

## lncDILC Downregulation in Liver Cancer‐Associated Fibroblasts Drives Pro‐Invasive Conversion via a miR‐6071‐ZNF395 Axis

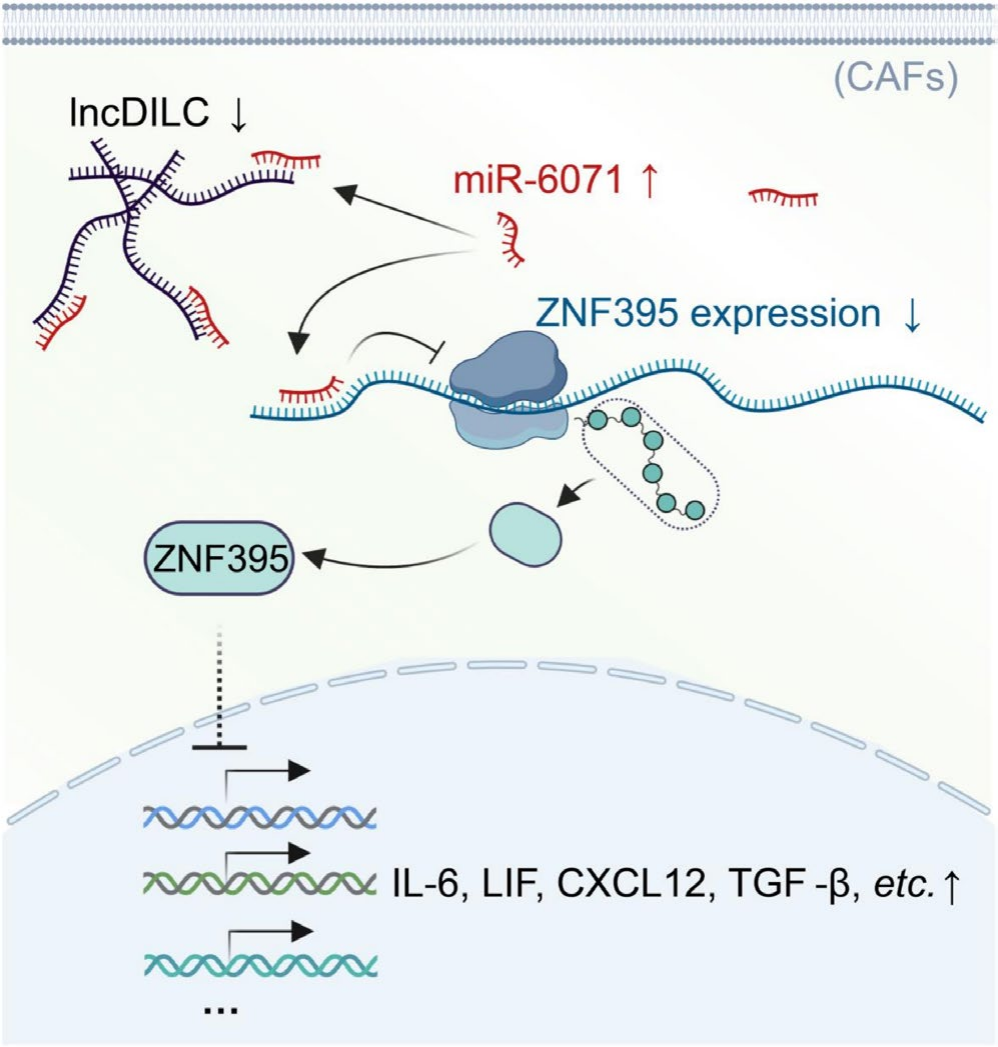

Liver cancer remains a major global health concern due to its aggressive nature and limited treatment options. One of the biggest challenges is its ability to spread beyond the primary tumor location to other organs and areas of the body, a process known as metastasis.

Recent evidence now suggests that metastatic progression can be influenced by the surrounding tumor environment. Among the cells found in the surrounding tumor environment, fibroblasts, normally responsible for tissue repair and maintenance, can be reprogrammed into cancer‐associated fibroblasts (CAFs), which actively support tumor growth and progression. Understanding how this transformation occurs is critical for developing better treatment strategies.

This study focuses on a molecule called lncDILC, a type of RNA that helps regulate gene expression. According to the authors, the levels of lncDILC were significantly reduced in fibroblasts located near liver tumors. They also reported that lower levels of lncDILC led to fibroblasts undergoing major behavioral changes, including the release of chemicals that promoted liver cancer cell migration, invasion, and tumor formation—a clear shift toward a cancer‐promoting role.

The study also identified the pathway behind this process. Reduced lncDILC increased the activity of miR‐6071, a small RNA molecule that suppresses ZNF395, a gene that normally helps maintain more stable fibroblast functions. When ZNF395 levels dropped, fibroblasts developed CAF‐like features that strengthened tumor progression. Restoring lncDILC levels prevented this harmful transformation and reduced cancer cell invasiveness.

Taken together, these findings reveal a key regulatory process that governs fibroblast behavior in the liver tumor environment. Disruption of this process promotes the conversion of fibroblasts into cancer‐supportive cells, contributing to disease progression. Importantly, these results suggest that targeting lncDILC or its downstream effects could help limit cancer spread and improve treatment responses.

By offering new insight into how the tumor environment encourages cancer growth, this research contributes to the development of more effective therapies for liver cancer—a vital step toward improving survival and patient quality of life.

https://onlinelibrary.wiley.com/doi/full/10.1111/cas.70236

